# Supplementary material for: Early Oxygen Treatment Measurements Can Predict COVID-19 Mortality: A Preliminary Study
Source: Healthcare (Basel). 2022 Jun 20;10(6):1146. doi: 10.3390/healthcare10061146 (PMC9222284; doi:10.3390/healthcare10061146)
Supplement: Supplementary file 1 [file healthcare-10-01146-s001.zip › healthcare-1682489-supplementary.pdf]

1. Supplementary information

Supplementary figure legends

Figure S1. Death due to COVID-19 infection is dependent on the severity of the immune-inflammatory response and correlates with the OSS. Laboratory blood test results for (A) Percentage of neutrophils (NEU%), (B) Absolute number of Lymphocytes (LYM abs.), (C), Level of urea in the blood (Urea-B), and (D) Level of LDH in the blood (LDH-B) of the survivor and non-survivor groups 14 days post-admission. (E) Heatmap showing the laboratory blood tests and their correlation with the OSS for the survivor and non-survivor groups.

Supplementary tables

Table S1. Description of the features considered in the machine-learning model.

Type	Feature	Description
Medical history	Age	A numeric feature that indicates the age of the tested individual in years
	Existence of a background disease	A binary feature that indicates a pre-existing disease, such as anemia, COPD, dementia, or diabetes
	Gender	A binary feature that indicates the gender of the tested individual
OSS	OSS (max, mean, increase)	Three numeric features that indicate the maximum, average, and increase in the OSS value during hospitalization
Blood tests	LDH (min, max, mean, increase)	Four numeric features that indicate the minimum, maximum, average, and increase in LDH in the blood during hospitalization
	LYM% (min, max, mean, increase)	Four numeric features that indicate the minimum, maximum, average, and increase in LYM% in the blood during hospitalization
	CRP (min, max, mean, increase)	Four numeric features that indicate the minimum, maximum, average, and increase in CRP in the blood during hospitalization

Supplementary figures

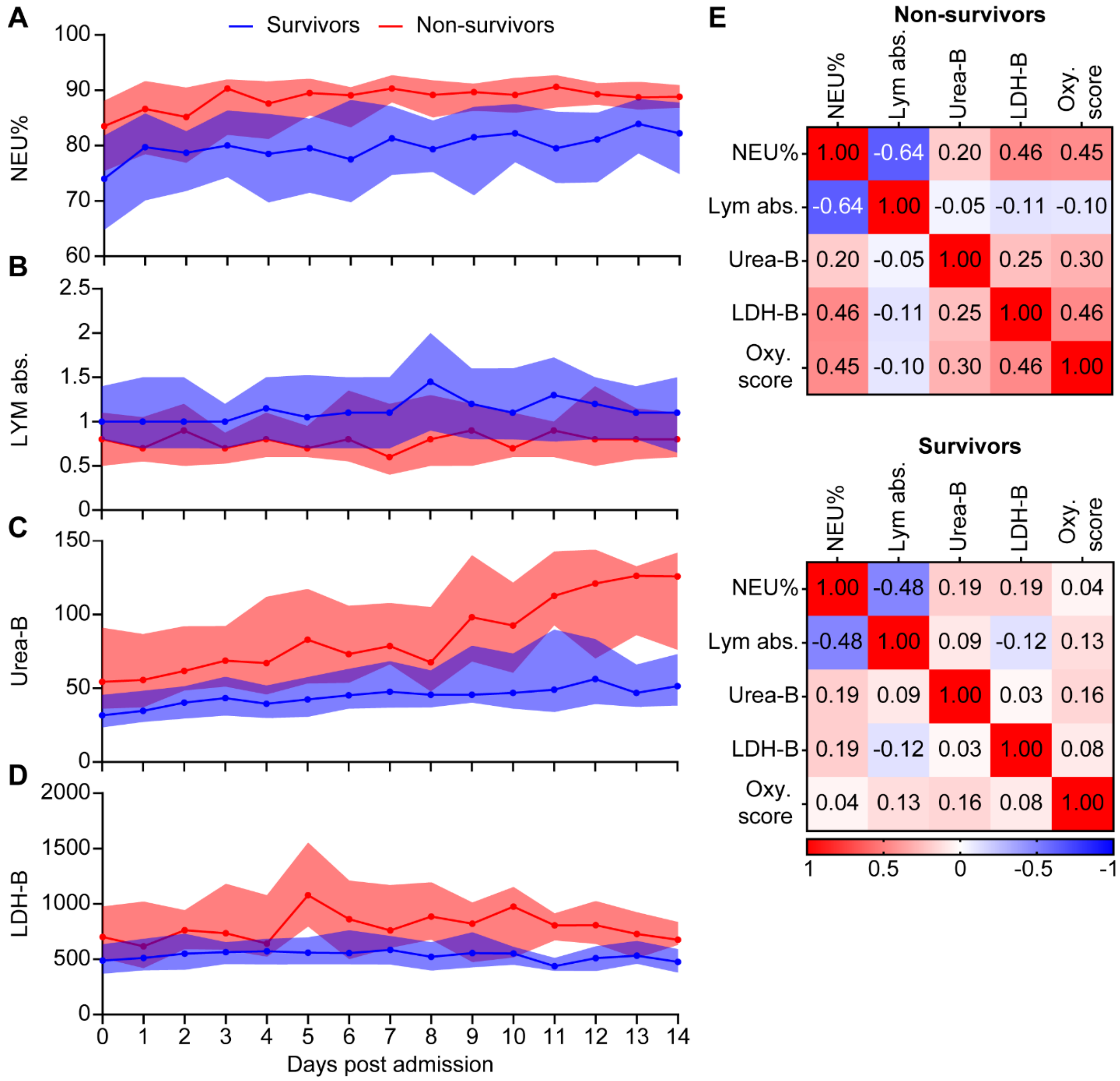

Figure S1. Death due to COVID-19 infection is dependent on the severity of the immune-inflammatory response and correlates with the OSS.
